# Facial onset sensory and motor neuropathy in a pain clinic outpatient: a case report

**DOI:** 10.1186/s13256-021-03212-7

**Published:** 2021-12-24

**Authors:** Hiroki Hanawa, Ryo Nagaoka, Yuya Fukuda, Kazuya Akutsu, Teppei Yamada, Shinsuke Hamaguchi

**Affiliations:** Department of Anesthesiology and Pain Medicine, Dokkyo Medical University School of Medicine, Kitakobayashi 880, Mibu, Shimotsuga-gun, Tochigi, 321-0293 Japan

**Keywords:** Facial onset sensory and motor neuropathy (FOSMN), Facial paresthesia, Cervical facet arthropathy, Cervical pain

## Abstract

**Background:**

Facial onset sensory and motor neuropathy is a very rare sensorimotor disorder characterized by facial onset and gradual progression, with approximately 100 cases reported worldwide in 2020. We report on our experience with a facial onset sensory and motor neuropathy case in our outpatient pain clinic.

**Case presentation:**

A 71-year-old Japanese man with a previous diagnosis of trigeminal nerve palsy complained of facial paresthesia, cervical pain, and arm numbness. Cervical facet arthropathy was diagnosed initially, but neither pharmacotherapy nor nerve blocking alleviated his symptoms. We suspected bulbar palsy based on the presence of tongue fasciculation, which prompted referral to a neurologist. Based on a series of neurological examinations, facial onset sensory and motor neuropathy was ultimately diagnosed.

**Conclusions:**

Pain clinicians must be mindful of rare diseases such as facial onset sensory and motor neuropathy; if they are unable to make a diagnosis, they should consult with other competent specialists.

## Background

Facial onset sensory and motor neuropathy (FOSMN) is characterized by facial-onset sensory disturbance that can later spread to the scalp, neck, trunk, and extremities; this stage is followed by motor neuron loss [1]. FOSMN is a very rare neurological disease, with about 100 cases reported worldwide as of 2020 [1]. Despite the diagnostic criteria that might be formulated based on known cases, the diagnosis of FOSMN is considered difficult even for veteran neurologists. In this report, we present a case of FOSMN to emphasize to pain specialists that knowledge regarding uncommon disease such as FOSMN is essential for differential diagnosis, and that they should refer these cases to relevant specialists if they are unable to make a definitive diagnosis.

## Case presentation

A 71-year-old Japanese man (height: 170.2 cm; weight: 54.5 kg) was referred to our outpatient clinic with the chief complaints of right facial numbness and itching, cervical pain, and right arm numbness with spasms in 2020. The patient’s history indicated that had been taking a calcium channel antagonist for hypertension (amlodipine, 5 mg/day) for the past 10 years. In 2016, he developed numbness and hypoesthesia in the right side of all branches of trigeminal nerve distribution and was diagnosed with idiopathic trigeminal nerve palsy. He has been taking gabapentin, amitriptyline, pregabalin, and clonazepam; however, these drugs did not improve his symptoms. In 2019, the patient developed cervical pain and right arm numbness; he was diagnosed with no evidence of a neurological abnormality.

At his first visit in 2020, he had complained of right facial numbness and itching, cervical pain, and right arm numbness. His medical records indicated a right facial palsy with mild numbness localized to the right cheek that might be gradually expanding. However, the sensory disturbance had not spread beyond the face at the time of the first visit.

A seemingly involuntary spasmodic twitching was noted at the right labial commissure. In addition, his speech was slightly slurred, and the right side of his mouth would fatigue with continued mastication, suggesting oral dyskinesia or a similar neurological disease. Right-dominant bilateral trapezius muscle tenderness and pain of 62 mm in visual analog scale were observed. The sites of the right arm numbness (rated 9/10) and triceps spasm were consistent with C6 involvement. Manual muscle testing was not performed at this time. Blood biochemistry results were within normal limits. On plain cervical radiographs, cervical straightening from C2 to C7 was observed in the lateral view, while signs suggesting stenosis of the right C6 intervertebral foramen were observed in the oblique view. Cervical magnetic resonance imaging revealed evidence of disc protrusion at C5/6 and at C6/7, with no apparent spinal compression or right C6 neuroforaminal stenosis.

These findings suggested a diagnosis of cervical discogenic pain or facet arthritis, and his facial symptoms suggested oral dyskinesia or another neurological disease, but not trigeminal nerve palsy. We prescribed mirogabalin (5 mg/day), extended-release paroxetine (12.5 mg/day), and clonazepam (0.5 mg/day) to be taken at bedtime, as well as biperiden (2 mg/day). However, this did not improve his symptoms after 1 week. For diagnostic and therapeutic purposes, we performed a stellate ganglion block at the second visit; however, this sympathetic nerve block also failed to improve his symptoms.

Because we observed tongue fasciculation while examining his complaints of dysphagia at his third visit, we diagnosed bulbar palsy as the cause of his dysphagia. We referred him to the neurology department, where an examination revealed evidence of hypoesthesia and muscle weakness in the right C6 distribution, tongue fasciculation, muscle weakness during neck extension, and exaggerated deep tendon reflexes. At the first visit to the outpatient service of neurology, the neurologist noted a progressive weight loss. Consequently, the neurological findings were as follows: sensory disturbance in the left trigeminal area and motor weakness on the left face, dysphagia, dysarthria, perioral fasciculation, tongue atrophy, and fasciculation. Attenuation of the corneal and blink reflexes as well as bulbar palsy were confirmed in a series of neurological tests. In the manual muscle strength test, weakness was observed in the trapezius, infraspinatus, and subscapularis muscle, but the muscle strength of the lower limbs was normal. The patient also had a positive Trömner reflex but showed no abnormalities in the Romberg test, suggesting upper motor neuron disease with pyramidal tract involvement. Moreover, magnetic resonance imaging of the head and neck showed atrophy of the tongue, trigeminal nerve, and masseter and temporalis muscles. Based on these collective findings, FOSMN was diagnosed, and a neurologist assumed responsibility for the patient’s treatment. The patient’s muscle weakness, weight loss, conversational difficulties, and dysphagia rapidly progressed following our encounters, necessitating a tracheotomy and gastrostomy.

## Discussion

First reported as a “syringomyelia-like” condition by Vucic *et al.* in 2006 [2], FOSMN is an exceedingly rare sensorimotor disorder characterized by the onset of facial sensory abnormalities and gradual progression. FOSMN occurs twice as often in men as in women, and has a mean age of onset of 54 years and a mean duration of 8.9 years [2]. Although the etiology of FOSMN has not yet been clarified, an association with amyotrophic lateral sclerosis is suggested based on the pathological study of transactivation response DNA binding protein 43 (TDP-43) [3]. It has been reported that progressive facial paresthesia is the first indication of the disease in approximately 91% of cases, and bulbar palsy occurs in 97% of cases [4]. Grudzińska *et al.* [5] reported that the bulbar symptoms, such as dysarthria and dysphagia, emerge in the second half of the disease’s course, along with attenuation/extinction of the corneal and blink reflexes.

We initiated treatment based on a suspected diagnosis of idiopathic trigeminal nerve palsy with cervical spondylotic changes at his first visit. However, tongue fasciculations combined with unresponsiveness to stellate ganglion blocking and pharmacotherapy suggested bulbar symptoms, which prompted us to refer him to the neurology department. According to a retrospective review of his medical history, facial paresthesia 5 years prior might have been the first manifestation of FOSMN; this evolved to muscle weakness in his head and neck, resulting in pain from facet arthropathy as he struggled to maintain head posture. He also suffered from C6 radiculopathy based on hypoesthesia in the right C6 dermatome and cervical instability, which was exacerbated by the same muscle weakness. Therefore, in the diagnosis of a rare disease such as FOSMN, these important points of a patient’s medical history might be reconsidered. The diagnosis was made because his physical and laboratory findings matched most of the clinical features of FOSMN described in the manuscript by Zheng *et al.* [4].

In addition, de Carvalho *et al.* described that a patient diagnosed with cramp-fasciculation syndrome presented with muscle spasms and fasciculation throughout the body in later stages of amyotrophic lateral sclerosis [6]. Therefore, his right arm spasms might have been a manifestation of cramp-fasciculation syndrome, according the evidence linking FOSMN with amyotrophic lateral sclerosis [5]. This question requires further detailed testing to resolve. Although we were able to diagnose FOSMN in the present case, we emphasize that an accurate assessment of a patient’s neurological findings at a pain clinic is very important to recognize rare diseases such as FOSMN. Moreover, it is important that we must be informed of the existence of such rare diseases and promptly consult with other specialists.

## Conclusion

We treated a patient in our outpatient service whose chief complaints were facial numbness and itching, cervical pain, and right arm numbness with spasms. Based on the results of a series of examinations for neuromuscular disorders, the patient was ultimately diagnosed with FOSMN, a very rare sensorimotor disorder. Pain medicine specialists must be cognizant of rare diseases such as FOSMN, and should refer such patients to other competent specialists if they are unable to make a definitive diagnose.

## Data Availability

The datasets used and/or analyzed during the current study are available from the corresponding author on reasonable request.

## Abbreviations

ALS: Amyotrophic lateral sclerosis
CFS: Cramp-fasciculation syndrome
FOSMN: Facial onset sensory and motor neuropathy
TDP-43: Transactivation response DNA binding protein 43
